# Supplementary material for: MetaRibo-Seq measures translation in microbiomes
Source: Nat Commun. 2020 Jun 29;11:3268. doi: 10.1038/s41467-020-17081-z (PMC7324362; doi:10.1038/s41467-020-17081-z)
Supplement: Supplementary file 10 — Supplementary Data 7 [file 41467_2020_17081_MOESM10_ESM.zip › File2/Confidence_High_Taxonomy/260494_out.krona.html]

Javascript must be enabled to view this page.

members
magnitude
magnitudeUnassigned
count
unassigned
taxon
rank

260494\_out

72

2
superkingdom
72

phylum
72
976

200643
72
class

171549
72
order

171550
1
family

1
genus
239759

1
species

SRS018984\_contig\_number\_3051
28117

family
1
2005525

1
genus
375288

46503

SRS147139\_contig\_number\_12189
1
species

1
family
171552

1
genus
838


SRS043768\_contig\_number\_30724
1
species
1262923

815
69
family

69

SRS012273\_contig\_number\_45443SRS013098\_contig\_number\_32820SRS017103\_contig\_number\_contig-100\_669.167670SRS018541\_contig\_number\_contig-100\_819.92552SRS018575\_contig\_number\_6646SRS020233\_contig\_number\_11004SRS023715\_contig\_number\_16451SRS054590\_contig\_number\_1377SRS058770\_contig\_number\_1324SRS063040\_contig\_number\_1101SRS078419\_contig\_number\_16731SRS098655\_contig\_number\_12368SRS098717\_contig\_number\_13224SRS1054691\_contig\_number\_contig-100\_1483.112612SRS1055043\_contig\_number\_26767SRS1055099\_contig\_number\_contig-100\_827.82065SRS143598\_contig\_number\_15737SRS149784\_contig\_number\_contig-100\_411.171973
18
816
genus

371601
species
1

SRS019286\_contig\_number\_contig-100\_505.49638


SRS013215\_contig\_number\_474SRS013940\_contig\_number\_14169SRS016018\_contig\_number\_4553SRS016381\_contig\_number\_contig-100\_661.35499SRS017701\_contig\_number\_902SRS017821\_contig\_number\_33135SRS018049\_contig\_number\_558SRS018133\_contig\_number\_2551SRS023346\_contig\_number\_791SRS024435\_contig\_number\_32212SRS024549\_contig\_number\_4482SRS024663\_contig\_number\_13139SRS042628\_contig\_number\_35572SRS045195\_contig\_number\_6466SRS045713\_contig\_number\_14406SRS048981\_contig\_number\_8113SRS057717\_contig\_number\_1388SRS075021\_contig\_number\_563SRS075341\_contig\_number\_10907SRS077024\_contig\_number\_12954SRS077127\_contig\_number\_180SRS101268\_contig\_number\_192SRS1041038\_contig\_number\_16722SRS1041112\_contig\_number\_2204SRS1041136\_contig\_number\_964SRS104636\_contig\_number\_992SRS1055034\_contig\_number\_1598SRS142712\_contig\_number\_3394SRS148511\_contig\_number\_11793SRS148874\_contig\_number\_16743SRS149879\_contig\_number\_27364
31
species
357276

species
5

SRS014613\_contig\_number\_3421SRS014923\_contig\_number\_36522SRS056519\_contig\_number\_2015SRS101433\_contig\_number\_187SRS1041145\_contig\_number\_757
2292935


SRS048060\_contig\_number\_2043
1
species
246787

1841855
1

SRS058070\_contig\_number\_24144
species

species
1

SRS140645\_contig\_number\_1196
1263056

5
species

SRS064232\_contig\_number\_803SRS065504\_contig\_number\_25794SRS077392\_contig\_number\_44167SRS147653\_contig\_number\_3548SRS148319\_contig\_number\_1264
821

1
species

SRS1055067\_contig\_number\_16192
2292939

818
1
species

SRS015890\_contig\_number\_11674

820
1

SRS052078\_contig\_number\_272
species

species
3

SRS020394\_contig\_number\_6007SRS020869\_contig\_number\_15982SRS043001\_contig\_number\_937
46506
